# Effect of Phantom Containing Implants Angulation on the Amount of Metal Artifacts Around the Implants in CBCT Images

**DOI:** 10.1155/ijod/8828788

**Published:** 2026-06-12

**Authors:** Solmaz Valizadeh, Mahkameh Moshfeghi, Zeinab Bahrani, Behzad Houshmand, Seyed Sepehr Mirebeigi-Jamasbi, Maedeh Asnaashari

**Affiliations:** ^1^ Department of Oral and Maxillofacial Radiology, School of Dentistry, Shahid Beheshti University of Medical Sciences, Tehran, Iran, sbmu.ac.ir; ^2^ Department of Prosthodontics, School of Dentistry, Shahid Beheshti University of Medical Sciences, Tehran, Iran, sbmu.ac.ir; ^3^ Department of Periodontics, School of Dentistry, Shahid Beheshti University of Medical Sciences, Tehran, Iran, sbmu.ac.ir; ^4^ Research Committee, School of Dentistry, Shahid Beheshti University of Medical Sciences, Tehran, Iran, sbmu.ac.ir; ^5^ Department of Oral and Maxillofacial Radiology, School of Dentistry, Hamadan University of Medical Sciences, Hamadan, Iran, umsha.ac.ir

**Keywords:** angulation, cone-beam computed tomography (CBCT), dental implant, metal artifact

## Abstract

**Objective:**

Artifacts from dental implants impact the clarity of cone‐beam computed tomography (CBCT) scans, posing challenges to radiographic detection in adjacent areas. The aim of this in vitro study is to examine the effect of angling a phantom incorporating implant fixtures on the amount of metal artifacts in CBCT scans.

**Materials and Methods:**

Three titanium fixtures, each with a diameter of 4.2 mm and a height of 12 mm were positioned within a bone block made of bovine mandibular bone. The specimen was placed on a surveyor table. The device was employed to regulate the rotation of the phantom along anterior–posterior (*α*) and right‐left (*β*) axes. For each axis, the sample was scanned at three angles (0°, 15°, and 30°), resulting in a total of 12 scans. Each scan includes 10 regions of interest (ROIs). The absolute gray values (GVs) of all ROIs were determined at two levels (apical and coronal), and the mean and standard deviation (SD) were calculated. Using SPSS version 23 software, two‐way ANOVA was employed to examine the impact of the angle and plane of rotation on the quantity of metal artifacts.

**Results:**

Higher ΔGV was seen as the angle increased in the alpha plane, while in the beta plane, the highest ΔGV occurred at 15°. At the apical level, mean ΔGV in the alpha plane (0.036) was higher than in the beta plane (0.007), whereas at the coronal level it was higher in the beta plane (0.186 vs. 0.087). Corresponding *p*‐values for placement angle, rotation plane, and interaction of angle in the rotation plane were 0.734, 0.519, and 0.811 at the apical level and 0.254, 0.448, and 0.305 at the coronal level.

**Conclusion:**

Altering the implant placement angle and rotation plane did not significantly impact the quantity of metal artifacts in CBCT images.

## 1. Introduction

The essential need for high‐quality imaging in dental practices is undeniable for the precise diagnosis and treatment of oral and dental diseases. With the increasing popularity of dental implants as a reliable method for rehabilitating edentulous spaces, imaging has become even more essential for anticipating and preventing complications during and after implant placement [1, 2]. Among the three‐dimensional imaging modalities available in dentistry, cone‐beam computed tomography (CBCT) and conventional CT are the most widely used, offering detailed visualization of maxillofacial structures [3–5].

Compared with conventional CT, CBCT has become the preferred imaging modality in dental practice due to several advantages, including lower cost, smaller equipment size, faster scanning times, robust reconstruction software, sub‐millimeter spatial resolution, reduced patient radiation exposure, and the generation of isotropic voxels [6–9]. These advantages have made CBCT particularly valuable in multiple dental specialties such as implantology, endodontics, oral surgery, and orthodontics, where precise imaging is critical for diagnosis and treatment planning [2, 5, 10–15].

Despite these advantages, CBCT is prone to image degradation caused by artifacts, particularly metal artifacts generated by dental implants. These artifacts can impair image quality, challenging the accurate assessment of surrounding areas, potentially leading to diagnostic errors and compromised treatment outcomes [2, 12, 15]. In addition to metal artifacts, CBCT images may also be affected by ring artifacts, partial volume effects, and motion‐related artifacts [2, 16]. However, the presence of metallic materials, such as dental implants or restorative components, is especially problematic, as they absorb and scatter X‐rays. Consequently, CBCT images derived from these perturbed X‐rays exhibit disparities between the actual anatomy and the reconstructed image, often appearing as alternating bright and dark streaks that obscure clinically relevant details [2, 15, 17].

To address these challenges, previous studies have explored strategies aimed at reducing artifact formation. These include both hardware‐related approaches during image acquisition, such as adjusting exposure parameters, including voltage, voxel size, tube current, the number of base images, and field of view (FOV) size, and software‐based postprocessing techniques designed to suppress or filter artifacts. While specialized software tools have shown promise in reducing metal artifacts, they can sometimes inadvertently remove valuable anatomical information along with unwanted noise, limiting their clinical reliability [18–21]. Recent research has also begun to investigate how implant positioning and angulation within the FOV influence artifact expression. For example, one study reported that modifying the anterior–posterior angulation of implants reduced interimplant artifacts in CBCT images, whereas alterations in other spatial directions did not significantly affect artifact intensity [2].

The aim of this in vitro study is to examine the effect of angling a phantom incorporating implant fixtures on the amount of metal artifacts caused by dental implants. We hypothesize that varying implant angulation influences artifact formation and that optimizing this parameter can improve CBCT image quality in clinical practice.

## 2. Materials and Methods

### 2.1. Ethical Approval

This study was approved by the research ethics committee of the Shahid Beheshti University of Medical Sciences (Ethics Number: IR.SBMU.DRC.REC.1401.018).

### 2.2. Sample Preparation

The current in vitro study utilized CBCT images derived from a phantom incorporating implants positioned at various planes and angles. To achieve this, three titanium dental implants, a bovine mandibular bone, and wax (as a soft‐tissue simulation) were used. To conduct this investigation, a phantom containing an implant was imperative. To craft the designated phantom, the bovine mandibular bone was cut using a cooling water‐equipped cutting machine (Dorsa, Tehran, Iran), forming a rectangular cube with dimensions of 10.7 mm in width, 30 mm in length, and 20 mm in height. The dimensions of the bone block were measured using a ruler with an accuracy of 1 mm. The bony cube exhibited cortical bone layers on its buccal and lingual surfaces, with trabecular bone present in its central region.

**Figure 1:**
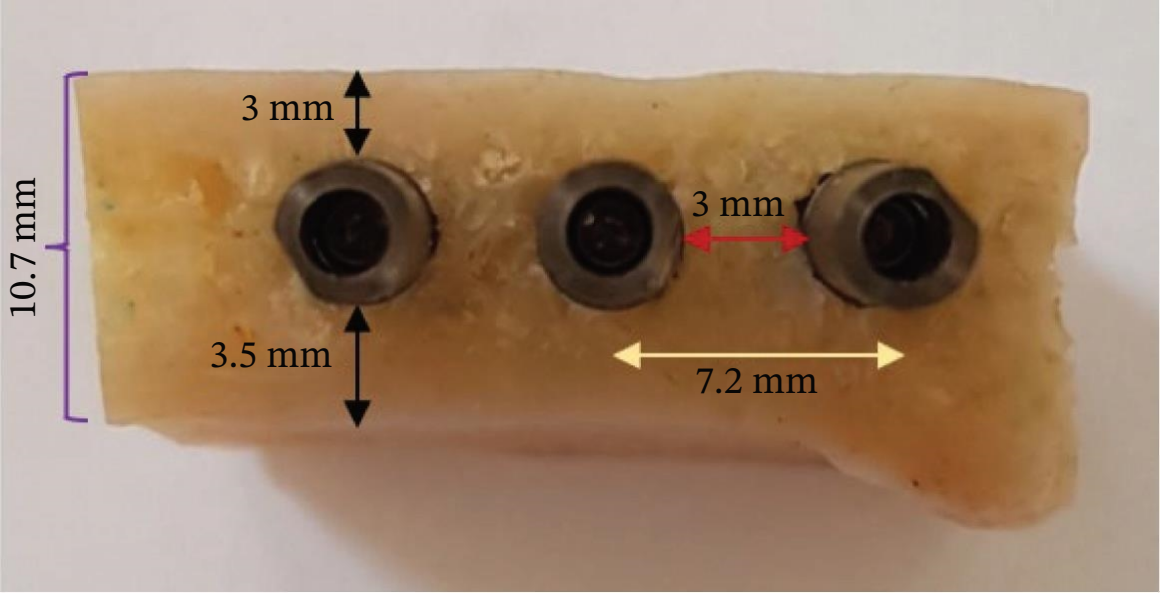
Bone block dimensions and distance between the implants.

Subsequently, three titanium fixtures (Implants Diffusion International [IDI], Montreuil, France), each with a diameter of 4.2 mm and a height of 12 mm, along with three titanium abutments (IDCAM‐straight abutment, IDI, Montreuil, France), were positioned within the prepared bone block under the guidance of a periodontist, utilizing the IDI implant kit. The spacing between each implant and the adjacent implant was established at 3 mm, resulting in a center‐to‐center distance of 7.2 mm between each consecutive pair of implants. A 3 mm layer of bone was present on the buccal side, while a 3.5 mm layer of bone surrounded each implant on the lingual side (Figure 1).

According to the research of Schropp et al. [22], simulating the average human soft tissue in laboratory imaging studies involves using a wax layer with a thickness ranging from 13–17 mm. In this study, a 13 mm thick layer of pink rose wax (Polywax, Modeling wax, Izmir, Turkey) was applied to cover the implant.

**Figure 2:**
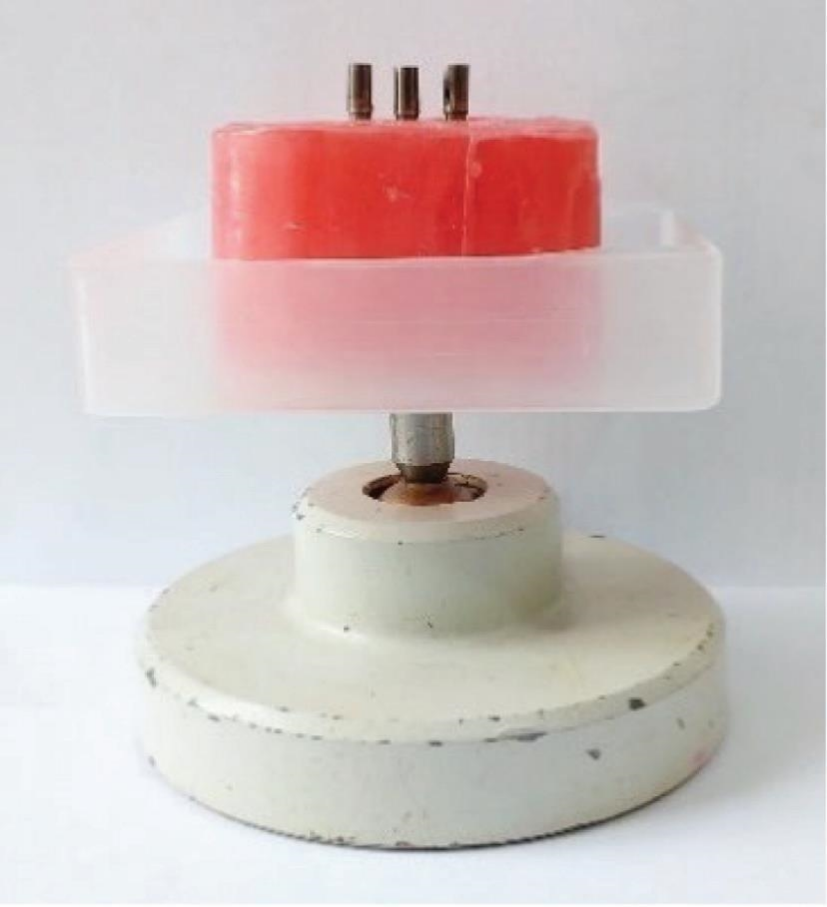
The phantom placed on the surveyor table.

**Figure 3:**
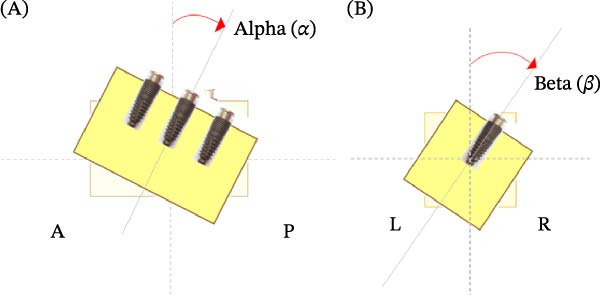
Schematic illustration of phantom rotation planes. (A) Anterior–posterior axis (alpha plane). (B) Right‐left axis (beta plane). A, anterior; L, left; P, posterior; R, right.

A surveyor table was employed for imaging the implants at various angles. Recognizing the potential for artifacts due to the metallic nature of the surveyor table and to prevent interference with the images, the upper segment of the table was substituted with a polypropylene rectangular container. The container had a thickness of 1.5 mm, measured using a caliper (3DJAKE Caliper, Vienna, Austria) with an accuracy of 0.02 mm. Its dimensions included sides measuring 7 cm × 8 cm, and the walls had a length of 2 cm, determined with a ruler with an accuracy of 1 mm. Subsequently, the prepared phantom was securely fixed within the mentioned container using drop glue (Razi Super Glue Instant Bond Adhesive, Iran) (Figure 2). The device was employed to regulate the rotation of the phantom along the two spatial axes. The rotation along the anterior–posterior axis was denoted as alpha (*α*) rotation, while the rotation along the right‐left axis was referred to as beta (*β*) rotation (Figure 3). The angles were determined by situating the surveyor table on a sheet featuring an image of a circle divided into 360 parts.

### 2.3. CBCT Scanner and Exposure Parameters

To assess the stability of mA and kVp, a routine daily check of the device was executed prior to the scans. Then, the prepared phantom was positioned in the VGi CBCT New Tom scanner (Quantitative Radiology, Verona, Italy), and CBCT scans were acquired under consistent exposure conditions, adhering to the device standards, as outlined below:

FOV size: 8 mm × 8 mm/Voltage: 110 kVp/exposure time: 3.6 s/beam current: 2.56 mA.

For scan preparation, the phantom was situated on the plastic plate designated for phantom positioning within the CBCT machine. The FOV size was consistently maintained across various scans, and the phantom’s position was meticulously adjusted using the reference laser lights of the scanner device. These lasers, designed for precise positioning, ensured that the phantom was centered within the FOV.

### 2.4. Study Groups

For each *α* and *β* rotation, the sample was positioned at three distinct angles (0°, 15°, and 30°), resulting in a total of 12 scans conducted, as outlined below:

1. Scan of the phantom without implants (control group) in the alpha plane and 0° angle.
2. Scan of the phantom without implants (control group) in the alpha plane and 15° angle.
3. Scan of the phantom without implants (control group) in the alpha plane and 30° angle.
4. Scan of the phantom without implants (control group) in the beta plane and 0° angle.
5. Scan of the phantom without implants (control group) in the beta plane and 15° angle.
6. Scan of the phantom without implants (control group) in the beta plane and 30° angle.
7. Scan of the phantom containing the implants (experimental group) in the alpha plane and 0° angle.
8. Scan of the phantom containing the implants (experimental group) in the alpha plane and 15° angle.
9. Scan of the phantom containing the implants (experimental group) in the alpha plane and 30° angle.
10. Scan of the phantom containing the implants (experimental group) in the beta plane and 0° angle.
11. Scan of the phantom containing the implants (experimental group) in the beta plane and 15° angle.
12. Scan of the phantom containing the implants (experimental group) in the beta plane and 30° angle.

It is important to note that for beta‐plane acquisitions, the phantom was rotated by 90° relative to the alpha‐plane configuration to ensure distinct scanning geometries.

### 2.5. Image Quantitative Assessment

**Figure 4:**
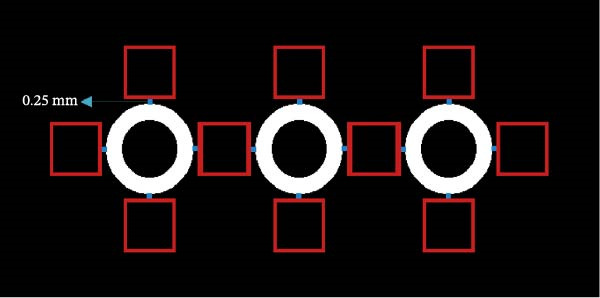
Schematic axial CBCT image illustrating implants (white circles) and ROIs (red squares).

Utilizing the OnDemand 3D Application image processing software, Version 10.0.1 (Cybermed, Seoul, Korea), each scan was saved in the DICOM (Digital Imaging and Communications in Medicine) standard format. Subsequently, the aforementioned software was employed for the quantitative measurement of the artifact quantity. To measure the gray values (GVs) in the regions of interest (ROIs) within the apical section of the implant, we accessed the 3D module in the OnDemand software. To enhance measurement accuracy, a 1.5x filter was initially activated across all coronal, sagittal, and axial sections. Following the approach from prior research [2], in the axial section, we configured the thickness to 1 mm (TH: 1 mm). We identified three points, aligning them with the lowest part of the implant. Following that, we aligned the horizontal blue marker in the sagittal section to ensure that it passed through the three specified points. Subsequently, the horizontal blue marker was set so that the lowest part of this marker passed through the three mentioned points, ensuring that it was perpendicular to the surface of the three implants and aligned with the most apical part of the implants. After applying these settings, the corresponding axial CT slice was identified. In this slice, a straight line with a width of 0.25 mm was drawn perpendicular to the two adjacent implants, and then, a square ROI with a side length of 2.5 mm was positioned at the midpoint between the two implants (Figure 4). This procedure was repeated for all angles in the same manner, ensuring that the ROIs were consistently located in the same defined position across all scans.

**Figure 5:**
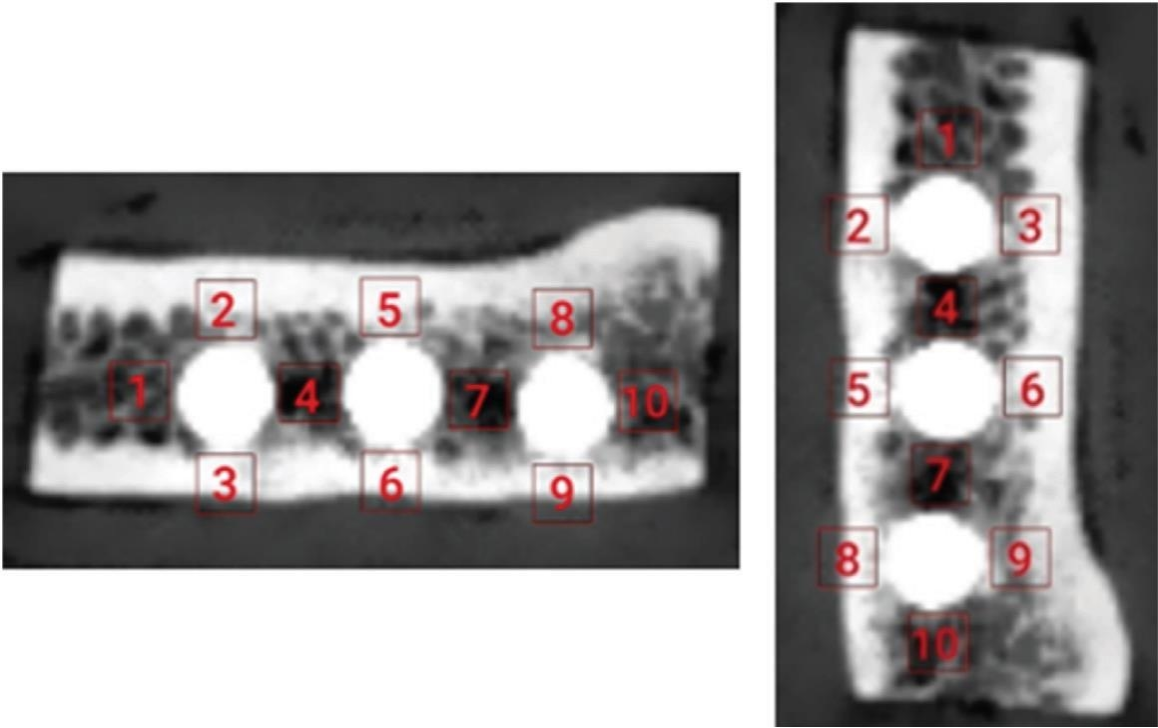
Selected ROIs defined on a single scan and displayed in different orientations to represent the (*α*) alpha and (*β*) beta analytical planes for visualization purposes.

In accordance with a previous study [2], we chose a rectangle from the various ROI shapes, and a square ROI with a side length of 4.7 mm was drawn. Each scan included 10 ROIs, all of which were defined on a single scan and are shown in different orientations to represent the alpha and beta analytical planes (Figure 5).

To quantify the GV in the ROIs at the coronal part of the implant, a procedure similar to that at the apical part was followed. However, four points were marked, aligning them with the uppermost part of the bone. Subsequently, the horizontal blue marker in the sagittal section was set so that the highest part of this marker passed through the four mentioned points. This adjustment positioned the marker perpendicularly to the surface of the three implants, effectively aligning it with the bone crest. Another difference was that the side length of the square‐shaped ROI in the coronal part was set at 2.5 mm, aligning with the dimensions specified in a previous study [2]. In that study, the ROIs were positioned ~0.3 mm from the implant surface [2]. Considering that the implants used in the present study were conical, with interimplant distances of 3 mm coronally and 5.2 mm apically, the ROI dimensions were decided to be 2.5 mm coronally and 4.7 mm apically. Therefore, the ROI margins remained ~0.25 mm away from the implant surface in both sections. In the previous study, the ROIs were defined in the axial section at least 0.3 mm away from the implant surface [2]. Although that study did not specify the rationale for this distance, it was likely to minimize the influence of blooming artifacts that occur adjacent to high‐density materials such as implants. Direct contact between the ROI and the implant surface could have made the GV measurements unreliable as the artifact margin would obscure the true boundary of the implant. In the present study, the ROI dimensions were determined accordingly: in both the coronal and apical sections, each ROI was designed to be 0.5 mm smaller than the exact distance between two adjacent implants, thereby maintaining an approximate 0.25 mm clearance from the implant surface on either side. This ensures that all measurements were obtained in the appropriate regions.

The absolute GVs of each of the 10 specified ROIs were determined in each scan at two distinct levels (apical and coronal) across all scans. For each ROI, the mean and standard deviation (SD) were calculated and documented in the checklist.

To establish intrarater reliability, 10 areas were assessed at 2‐week intervals, and the intraclass correlation (ICC) was computed. Additionally, throughout the study, any instances of ambiguity were addressed by consulting with an experienced oral and maxillofacial radiologist, ensuring clarification and resolution.

### 2.6. Statistical Analysis

The statistical analysis of the data was conducted using SPSS version 23 software. Descriptive data were presented quantitatively, including the mean and SD. Two‐way ANOVA was employed to examine the impact of the angle and plane of rotation on the quantity of metal artifacts in the region surrounding the implants in CBCT images. The probability values below 0.05 were considered statistically significant.

## 3. Results

In the 2‐week evaluation, the obtained ICC was equal to 98.5%, which indicates a high level of reliability in the assessment.

Table 1 presents the mean and SD of GVs at defined angles and planes at both apical and coronal implant levels.

**Table 1:** Gray value’s mean and SD by plane, degree, and level.

Group	Plane	Degree	Mean (apical)	Mean (coronal)	SD^a^ (apical)	SD (coronal)
Experimental	*α*	0	1373.54	1084.80	1255.88	877.82
		15	1362.10	992.98	1170.59	882.9
		30	1367.21	713.43	1145.07	965.54
	*β*	0	1367.39	974.02	1265.92	907.24
		15	1377.36	956.60	1269.17	1026.11
		30	1332.55	1161.70	1239.25	1021.82
Control	*α*	0	1360.88	985.42	1314.59	821.63
		15	1316.94	1110.75	1185.59	854.74
		30	1291.74	1147.39	1204.86	804.70
	*β*	0	1339.13	886.33	1303.73	815.00
		15	1329.56	1106.58	1254.72	849.14
		30	1325.67	1328.04	1232.30	944.31

^a^SD: standard deviation.

For statistical computations, the average GV difference (ΔGV) between the GV of each ROI (GV_ROI_) and the GV of the corresponding ROI in the control group (GV_control_) was determined based on percentage using Equation (1).

(1) ΔGV=GVROI− GVcontrolGVcontrol.

Since metal artifacts can induce both light and dark lines, therefore, to ensure accurate information about ΔGV and valid inference findings, we followed the approach outlined in previous studies [2]. Accordingly, the absolute value of GVs was employed in the calculations, ensuring that all GVs became positive. Positive or negative ΔGV indicates a higher or lower GV compared to the corresponding region in the control scan.

Table 2 displays the mean and SD of ΔGVs at defined angles and planes at both apical and coronal levels of the implant.

**Table 2:** Mean and SD of ΔGVs (gray values) by level, plane, and degree.

Level	Plane	Degree	Mean	SD^a^
Apical	*α*	0	0.0020	0.13902
Apical	*α*	15	0.0313	0.09105
Apical	*α*	30	0.0759	0.11232
Apical	Total *α*	—	0.0364	0.11594
Apical	*β*	0	−0.0075	0.37789
Apical	*β*	15	0.0232	0.08293
Apical	*β*	30	0.0049	0.07212
Apical	Total *β*	—	0.0074	0.21177
Coronal	*α*	0	−0.0274	0.46123
Coronal	*α*	15	0.0759	0.66398
Coronal	*α*	30	−0.3054	0.37535
Coronal	Total *α*	—	−0.0877	0.52481
Coronal	*β*	0	−0.0137	0.40158
Coronal	*β*	15	−0.2887	0.45381
Coronal	*β*	30	−0.2399	0.41751
Coronal	Total *β*	—	−0.1865	0.42737

^a^SD: standard deviation.

This study utilized two‐way ANOVA to examine the influence of implant placement angle and rotation plane on metal artifacts in CBCT images, considering multiple independent variables. The analysis explored the effects of implant placement angle, rotation plane, and their interaction on artifact quantity. Table 3 presents the results, evaluating the significance of changes in implant placement angle and rotation plane on the quantity of artifacts caused by dental implants in CBCT images.

**Table 3:** Two‐way ANOVA results for metal artifact formation on the apical and coronal level of the implants by placement angle and rotation plane.

Level	Factor	*p*‐Value
Apical	Degree	0.734
Apical	Plane	0.519
Apical	Degree × plane	0.811
Coronal	Degree	0.254
Coronal	Plane	0.448
Coronal	Degree × plane	0.305

The presented tables indicate that, at the apical level of the implant, the *p*‐values for the placement angle, rotation plane, and interaction of angle in the rotation plane were 0.734, 0.519, and 0.811, respectively. Similarly, at the coronal level, these values were 0.254, 0.448, and 0.305, respectively. Comparing these *p*‐values with the significance level of 0.05, it is observed that the assumption of significance for the effects of changes in the implant placement angle and rotation plane on the quantity of metal artifacts caused by dental implants in CBCT images is not confirmed at the 5% significance level.

Considering the nonsignificance of the interaction effect between the placement angle and rotation plane of the implants (*p*‐value = 0.811 at the apical level and *p*‐value = 0.305 at the coronal level), another two‐way analysis of variance test was conducted. In this analysis, the interaction effect was excluded to evaluate whether the impact of implant placement angle and rotation plane becomes significant after its removal. Following the removal of the interaction effect, at the apical implant level, the *p*‐values for implant placement angle and rotation plane were 0.721 and 0.507, respectively. At the coronal implant level, the *p*‐values for implant placement angle and rotation plane were 0.256 and 0.432, respectively. These values remained nonsignificant. Therefore, neither implant placement angle nor the rotation plane significantly affected the quantity of metal artifacts in CBCT images, suggesting no impact on image quality.

## 4. Discussion

This study aimed to investigate the influence of implant angulation on the amount of metal artifacts in CBCT images. The metal artifact caused by dental implants in CBCT images results in diminished image quality and distortion of adjacent anatomical structures, which can impact the dentist’s clinical judgment across different stages of diagnosis, treatment planning, and implant follow‐up [2]. The findings revealed that changing the implant angle or rotation plane (*α* or *β*) did not significantly affect the quantity of artifacts. These outcomes differ from those reported in previous investigations, particularly by Min and Kim [2] and Luckow et al. [23], which demonstrated that increasing the implant angle could reduce metal artifacts and improve image quality.

In a study by Min and Kim [2], the effect of seven different angles (0°, 15°, 30°, 45°, 60°, 75°, and 90°) across three rotational planes (*α*, *β*, and *γ*) was examined using a CBCT scanner (Alphard 3030). The results indicated that an increase in the *α*‐plane angle reduced metal artifacts in the interimplant region, whereas changes in the *β* and *γ* planes had minimal impact. However, that study used phantoms made of homogeneous polyvinyl siloxane impression material [2], which does not accurately simulate the heterogeneous anatomy of the human jawbone. In contrast, the present research employed bovine mandibular bone blocks, which more closely mimic the complex structure of the human bone [24–28]. This difference in phantom material is likely one of the main reasons for the conflicting findings, as the scattering and beam‐hardening behaviors differ between uniform and anatomical bone structures. Furthermore, Min and Kim analyzed 13 VOIs (at a single level along the implant’s longitudinal axis, with a depth of 2–3 mm from the crest) [2], while the present study evaluated 20 ROIs at two different implant levels (apical and coronal). Regarding the implant systems used, Min and Kim employed Point Implants [2], whereas this study used implants from IDI. Such variations in implant systems can affect artifact generation due to differences in X‐ray attenuation characteristics. Additionally, the imaging parameters used in Min’s experiment [2] (FOV 51 mm × 51 mm, 78 kVp, 17 s exposure time) differ substantially from those in the current research (FOV 8 mm × 8 mm, 110 kVp, 3.6 s exposure time). These differences in exposure conditions and device settings may have influenced artifact levels and image contrast, resulting in different outcomes.

In another study, Min and Kim [29] quantitatively evaluated artifacts based on microstructural parameters derived from micro‐CT and CBCT images. Their results again showed that an increased α‐plane angle reduced metal artifacts and enhanced the image quality. However, that investigation used artificial polyurethane bone blocks and a combination of micro CT (SkyScan 1076) and CBCT (Alphard 3030) scanners. The current study, instead, used a single CBCT unit (NewTom VGi), which operates at higher voltage and shorter exposure time. These differences in scanning devices, voxel sizes, and reconstruction algorithms could influence the expression of artifacts. Furthermore, Min and Kim [29] used parameters such as trabecular microstructure to quantify artifacts, whereas the present study adopted the SD of GV, which is a more direct and widely used CBCT‐based metric. Therefore, the variations in quantitative methodology could also contribute to the different findings.

Another comparable study by Luckow et al. [23] investigated mandibular tilts up to 14° and observed nearly a twofold improvement in image quality. Their work analyzed several rotational planes (*α*, *β*, and *φ*) using Straumann implants placed in pig mandible specimens. In addition, they utilized both micro‐CT (SkyScan 1172TM) and CBCT (3D Accuitomo 60) scanners along with a distinct reconstruction software (VG StudioMax) [23]. In comparison, the present study used bovine bone samples, IDI implants, and OnDemand software, with imaging performed using the NewTom CBCT system at 110 kVp and 3.6 s exposure. Differences in scanner type, voltage, exposure duration, and reconstruction algorithms can directly affect artifact magnitude and image noise. Moreover, Luckow et al. [23] calculated artifacts based on the discrepancy between CBCT gray levels and synchrotron radiation‐based μCT images, while the present study employed the SD of GVs. These methodological distinctions provide a reasonable explanation for the observed inconsistencies between the studies.

Regarding the impact of material type on artifact production, studies indicate that zirconium implants produce the highest artifacts, while titanium implants generate the least artifacts. Titanium‐zirconium implants exhibit an intermediate level of artifact production [21, 30, 31]. As titanium implants are currently the most prevalent choice for dental implants, given their favorable physical and chemical properties and biocompatibility [31], this study exclusively focused on investigating titanium implants.

Although numerous methods have been proposed for quantifying metal artifacts, no standardized approach has yet been established [20]. Mean and SD of GVs or contrast‐to‐noise ratio remain the most practical indices [19, 29, 32] as a decrease in SD alongside an increase in mean GV reflects a reduction in artifact intensity [33]. It should be noted, however, that the heterogeneous GV distribution in trabecular bone can affect the reliability of these measures [19]. Alternative approaches, such as the analysis of trabecular microstructural parameters, may provide a more accurate estimation of artifacts within the bone tissue [29].

Due to the variability in patient anatomy and exposure parameters, studies examining the amount of artifacts caused by dental implants in CBCT images often rely on laboratory investigations rather than patient image data. Laboratory studies, including the present research, offer precise control over variables, enabling the separate investigation of specific factors affecting the variable of interest. However, it is important to note that X‐ray beam interactions can vary among individual patients [34]. It is also worth noting that the laboratory setting of the present study eliminated the impact of motion‐related artifacts commonly encountered in clinical settings. As a result, these factors lead to the production of fewer artifacts compared to those observed in clinical environments [29]. It should be noted that the findings of the present study are specific to the scanning trajectory employed by the CBCT system used, which is based on a circular or limited‐angle circular acquisition geometry [35, 36]. Although implant plane rotation did not significantly influence metal artifact expression under these conditions, metal artifact formation in CBCT imaging is known to be multifactorial and influenced by acquisition geometry, exposure parameters, and object‐related factors [18–21, 37]. Previous studies have demonstrated that optimized or noncircular scanning trajectories can alter X‐ray beam paths and reduce beam hardening and scatter‐related artifacts [35, 36]. Therefore, the results of this study should not be generalized to CBCT systems employing alternative or optimized gantry trajectories, and the conclusions are limited to systems utilizing circular or limited‐angle circular trajectories similar to those used in the present investigation. Therefore, further clinical investigations are required to validate these findings under real‐patient scenarios.

## 5. Conclusions

Based on the results of the present research, altering the implant placement angle and rotation plane did not produce a statistically significant change in the amount of metal artifacts in the CBCT images. However, given the experimental nature of this study, using a bone block without simulating patient movement and testing only one CBCT unit and scanning protocol, these findings should be interpreted with caution. Further in vivo investigations involving various imaging systems and clinical conditions are required before drawing conclusions about the clinical effectiveness of these methods for reducing metal artifacts.

## Author Contributions

**Solmaz Valizadeh and Mahkameh Moshfeghi**: conceptualization, methodology, formal analysis, writing – review and editing, supervision. **Zeinab Bahrani**: methodology, writing – review and editing. **Behzad Houshmand**: methodology, resources, writing – review and editing. **Seyed Sepehr Mirebeigi-Jamasbi**: formal analysis, writing – original draft, visualization. **Maedeh Asnaashari**: conceptualization, methodology, investigation, data curation, visualization, writing – review and editing, project administration.

## Funding

The authors state that this project has not received any funding.

## Disclosure

All authors have read and approved the final version of the manuscript.

## Conflicts of Interest

The authors declare no conflicts of interest.

## Data Availability

The data that support the findings of this study are available from the corresponding author upon reasonable request.
